# Historical Hot Summers

**Published:** 1884-11

**Authors:** 


### HISTORICAL HOT SUMMERS.

PEOPLE who are already complaining of the heat while this summer is scarcely a month old says the *XIXeme Siecle*, should remember the extroardinary weather of former years. In 627 the heat was so great in France and Germany that all springs dried up ; water became so scarce that many people died of thirst. In 870 work in the fields had to be given up ; agricultural laborers persisting in their work were struck down in a few minutes, so powerful was the sun. In 903 the sun's rays were so fierce that vegetation burned up as under the action of fire. In 1000 rivers ran dry under the protracted heat, the fish were left dry in heaps and putrefied in a few hours. The stench that ensued produced the plague. Men and animals venturing in the sun in the summer of 1022 fell down dying, the throat parched to a tinder and the blood rushing to the brain. In 1132 not only did the rivers dry up, but the ground cracked on every side and became baked to the hardness of stone. The Rhine in Alsace nearly dried up. Italy was visited with terrific heat in 1139 ; vegetation and plants were burned up. During the battle of Bela in 1260 there were more victims made by the sun than by weapons ; men fell down sunstruck in regular rows. The summer of 1277 was also severe ; there was an absolute dearth of forage. In 1303 and 1304 the Rhine, Loire and Seine ran dry. In 1615 the heat throughout Europe became excessive. Scotland suffered particularly in 1625 ; men and beasts died in scores. The heat in several departments during the summer of 1705 was equal to that in a glass furnace. Meat could be cooked by simply exposing it to the sun. Not a soul dare venture out between noon and 4 p. m. In 1718 many shops had to close ; the theatres never opened their doors for several months. Not a drop of water fell during six months. In 1753 the thermometer rose to 118°. In 1779 the heat of Bologna was so great that a great number of people were stifled. There was not sufficient air for the breath, and people had to take refuge under ground. In July, 1793, the heat became intolerable. Vegetables were burned up and fruit dried upon the trees. The furniture and woodwork in dwelling-houses cracked and split up ; meat went bad in an hour. The rivers ran dry in several provinces during 1811 ; expedients had to be devised for the grinding of corn. In 1822 a protracted heat was accompanied by storms and earthquakes; during the drought legions of mice overran Lorraine and Alsace, committing incalculable damage. In 1832 the heat brought about cholera in France ; 20,000 persons fell victims to the visitation in Paris alone. In 1846 the thermometer marked 125 degrees in the sun. Finally, the summers of 1859, 1860, 1869, 1870, 1874, etc., although excessively hot, were not attended by any disaster.—*London Standard.*

